# Supplementary figures and images for: On the Prospect of Identifying Adaptive Loci in Recently Bottlenecked Populations
Source: PLoS One. 2014 Nov 10;9(11):e110579. doi: 10.1371/journal.pone.0110579 (PMC4226487; doi:10.1371/journal.pone.0110579)

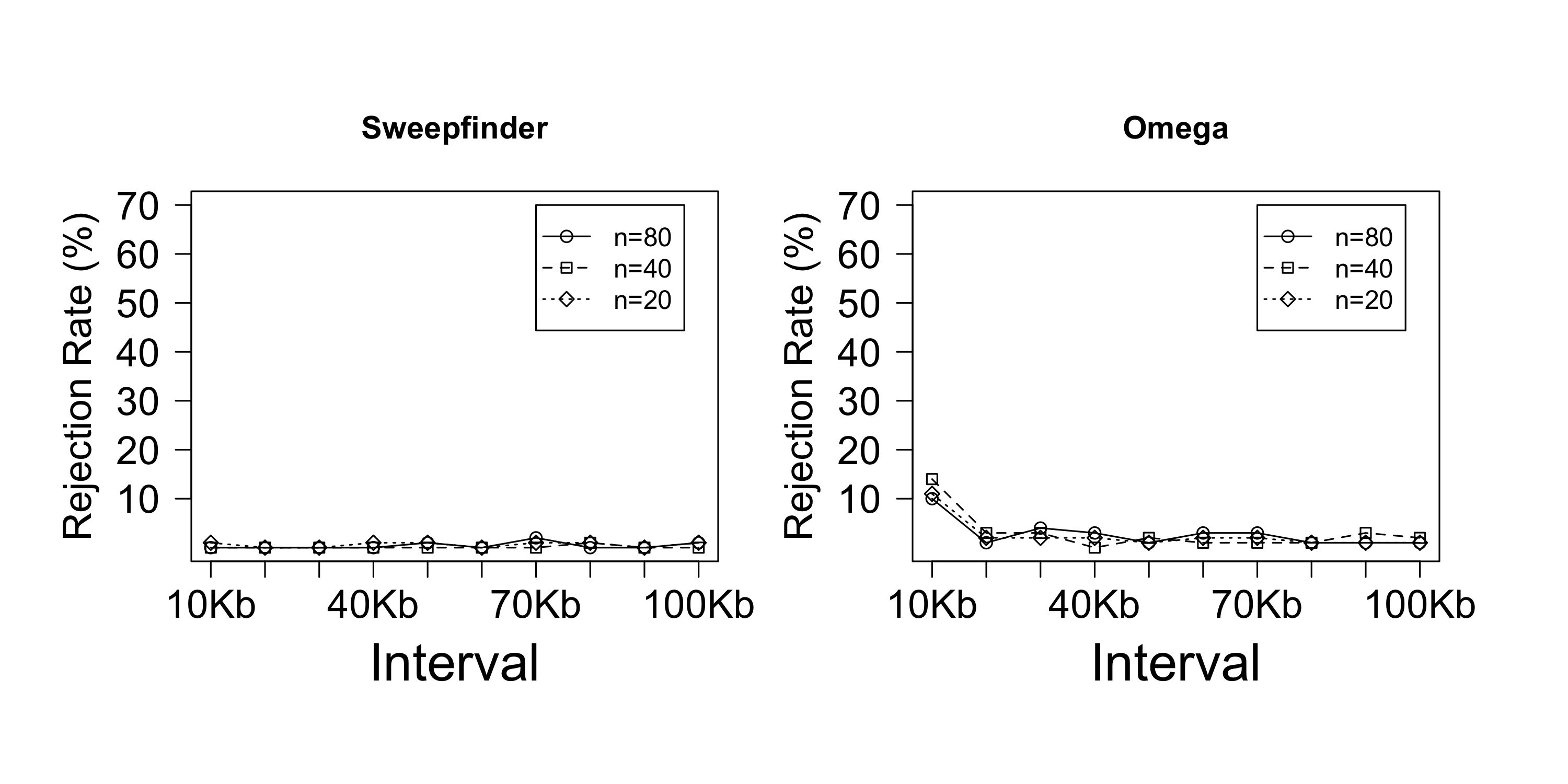

Supplement: Figure S1 — The fraction of simulated replicates rejecting the neutral model by Sweepfinder, with varying sample size. The simulations with demographic models mimic the history of Nebraska Sand Hills mice (N e = 50,000), and the time of the bottleneck (t r = 0.1) and time since fixation (τ = 0.1) and selection strength (s = 0.1) are fixed but sample size varies from N = 20 to 80. Ideal performance would be indicated by all replicates showing a significant signal at very small window sizes, suggesting an ability to localize the target. (PNG) [file pone.0110579.s001.png]

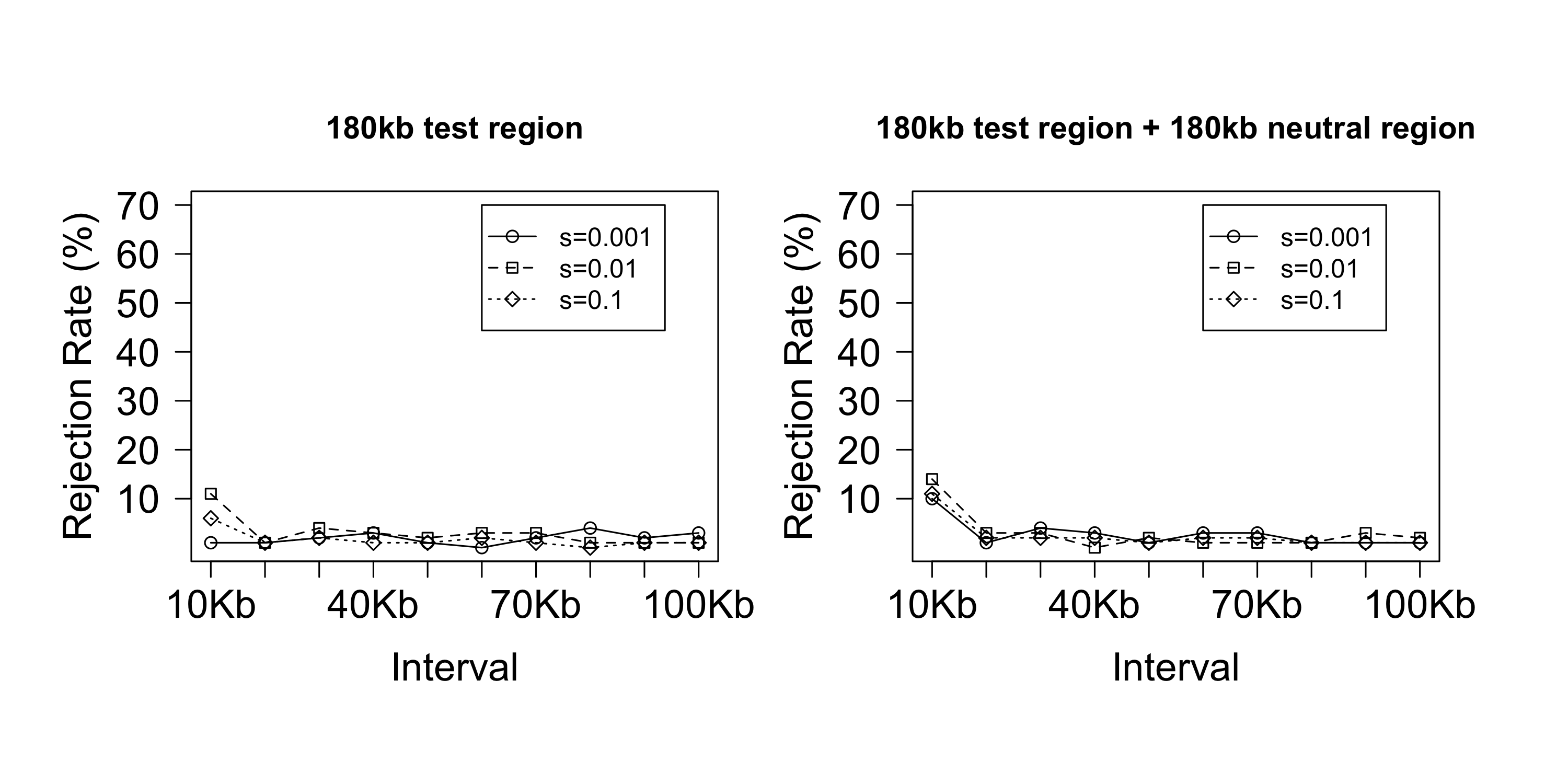

Supplement: Figure S2 — The fraction of simulated replicates rejecting the neutral model by Sweepfinder, with and without another 180kb simulated neutral region. The simulations 180kb with demographic models mimic the estimated history of Nebraska Sand Hills mice (N e = 50,000), the time of the bottleneck (t r = 0.1), the time since fixation (τ = 0.1), but selection strength varies from s = 0.001 to 0.1. The right panel shows the Sweepfinder performance with another 180kb simulated neutral regions with the same demographic parameters added. The results suggested that Sweepfinder could gain more efficacy in identifying sweeps with more neutral SNPs to build the background SFS, but the improvement is modest. (PNG) [file pone.0110579.s002.png]

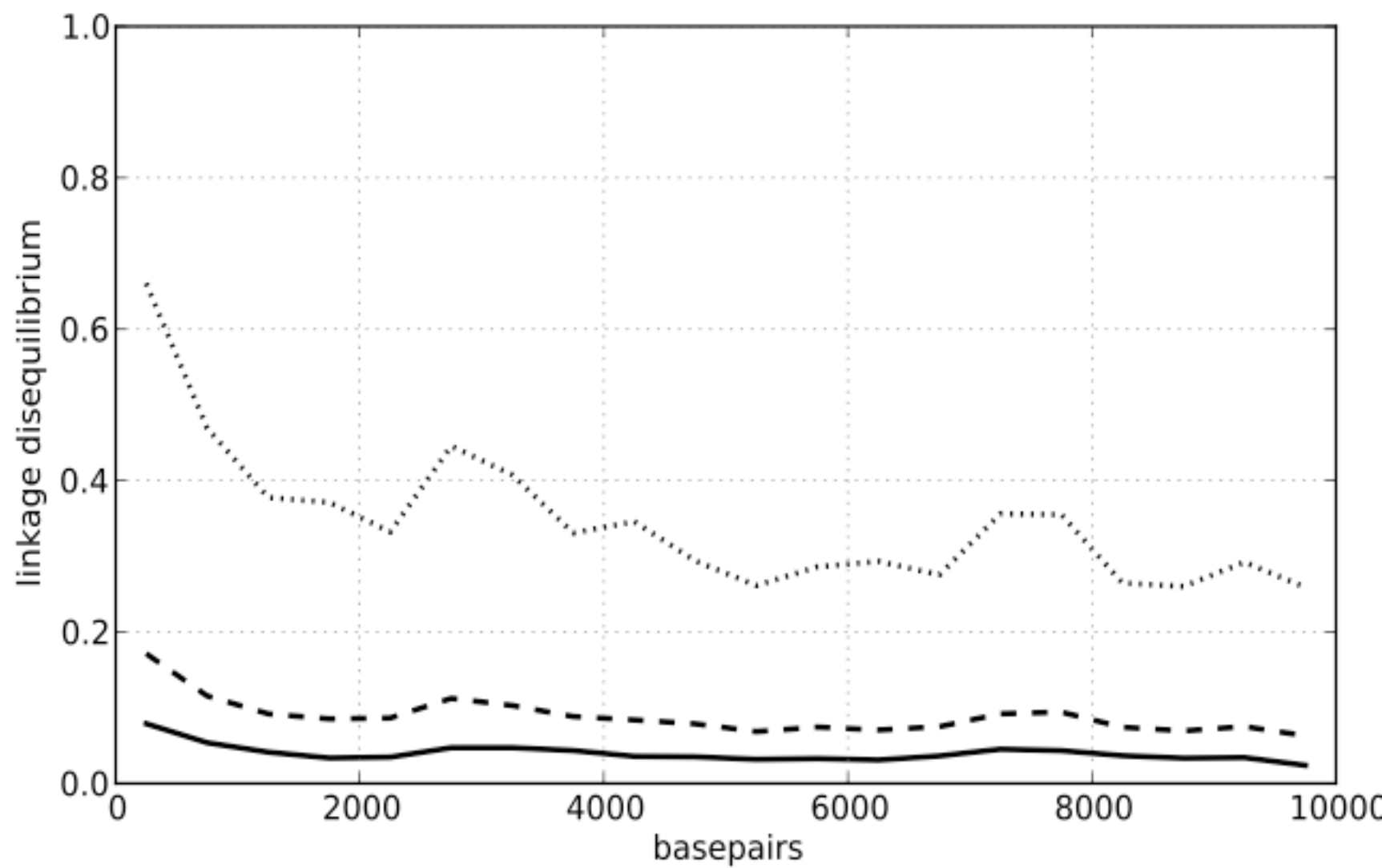

Supplement: Figure S3 — Decay of linkage disequilibrium (LD) as a function of physical distance between variable sites. In all panels the solid lines represent medians for each X axis category (physical spacing bin) centered on the plotted X coordinate, dashed lines represent means of spacing bins, and dotted lines represent 95th percentiles of spacing bins. (PDF) [file pone.0110579.s003.pdf]
